# Exploring musical self-efficacy and performance anxiety in young violin learners: insights from mainland China

**DOI:** 10.3389/fpsyg.2025.1575591

**Published:** 2025-06-25

**Authors:** Jiayi Ou, Chao Qin

**Affiliations:** ^1^Department of Musicology, Faculty of Social and Human Sciences, Nova University of Lisbon, Lisbon, Portugal; ^2^School of Education, Yunnan Minzu University, Yunnan, China

**Keywords:** music learning self-efficacy, music performance self-efficacy, music anxiety, violin undergraduates, musical instrument education

## Abstract

**Introduction:**

Music self-efficacy (MSE) and music performance anxiety (MPA) are critical issues in music education. This study examines MSE, including both music learning self-efficacy (MLSE) and music performance self-efficacy (MPSE), as well as MPA, among undergraduate violin students in mainland China.

**Methods:**

Data were collected via an online survey distributed to students from 11 music conservatories, resulting in 254 valid responses.

**Results and discussion:**

The results revealed moderate levels of MLSE and MPSE, with no significant gender differences. Differences were observed across academic years, with lower-year students reporting higher MLSE and MPSE than upper-year students. Conservatory level also influenced MLSE and MPSE, with A-level students scoring higher than B- and C-level students. MPA levels were generally high, but no gender or academic year differences were observed. However, a U-shaped pattern of MPA across conservatory levels was identified, with A- and C-level students experiencing higher anxiety than B-level students. Notably, MLSE was positively associated with MPA, while MPSE showed a negative association, suggesting that the two types of self-efficacy play divergent roles in MPA. These findings indicate that while MLSE and MPSE are moderately correlated, they differentially influence violin students’ MPA.

## 1 Introduction

Music learning and performance are personal and complex, requiring ongoing self-evaluation and adaptation. Performers must refine their technical skills while managing psychological well-being, which is crucial to their success. Among the psychological factors, music self-efficacy (MSE) and music performance anxiety (MPA) are particularly significant (Papageorgi et al., 2010; González et al., 2017; MacAfee and Comeau, 2020). Music self-efficacy is crucial for fostering effective learning environments, maintaining persistence, and achieving high levels of proficiency (Martin, 2007; Patston and Osborne, 2016; Steyn et al., 2016). In contrast, music performance anxiety is a persistent form of anxiety that can negatively impact the quality of performances and, in severe cases, lead to performance avoidance (Kenny, 2011). MSE and MPA are closely intertwined, with MSE often acting as a regulator of MPA intensity (Ritchie and Williamon, 2011).

Some studies suggest a negative correlation between MSE and MPA, indicating that music learners with high self-efficacy experience lower anxiety during performances due to greater confidence in their abilities (Paliaukiene et al., 2018; Burns et al., 2020; Bersh, 2021). However, Demet (2017) argues that high self-efficacy can increase sensitivity to failure, potentially triggering higher performance anxiety. This contradictory finding suggests that the relationship between MSE and MPA is complex and influenced by multiple factors. The discrepancies may arise from differences in instruments, variations in the measurement of self-efficacy, and cultural differences in music education practices.

Given the profound impact of MSE and MPA on music education, this study specifically targets young music learners in mainland China—undergraduate violin students from various music conservatories. (1) The study examined the differences in students’ musical self-efficacy—divided into music learning self-efficacy (MLSE) and music performance self-efficacy (MPSE)—as well as music performance anxiety (MPA) levels, with regard to gender, academic year, and the ranking of the different conservatories. (2) It also explored the relationships between MLSE, MPSE, and MPA.

## 2 Literature review

### 2.1 Music self-efficacy

Self-efficacy refers to an individual’s subjective judgment of their ability to achieve a specific goal before taking action (Bandura, 1977, 1982, 1986). In music education research, music self-efficacy specifically refers to an individual’s belief in their ability to successfully complete tasks within the domain of music (Ritchie and Williamon, 2010; Zarza-Alzugaray et al., 2020).

Self-efficacy plays a crucial role in enhancing students’ music learning and optimizing their performance behaviors (Zelenak, 2024). There is a strong connection between students’ self-efficacy and their achievement. First, self-efficacy significantly influences students’ learning attitudes and efforts. Students with high self-efficacy are more likely to embrace challenges and adopt a positive attitude toward music practice. This positivity helps them sustain motivation in music learning, thereby improving their musical achievements. Second, achieving success in musical tasks further reinforces students’ self-efficacy, creating a positive feedback loop (Ritchie and Williamon, 2010).

Ritchie and Williamon (2010) suggest that even within the same domain, individuals may hold varying self-efficacy beliefs for different tasks. For example, there may be distinctions between self-efficacy for learning a specific task and self-efficacy for performing that task. Learning primarily focuses on acquiring skills and knowledge, supported by various self-regulated learning strategies, whereas performance is often focused on stage practice. As a result, they distinguish MSE into two types: music learning self-efficacy (MLSE) and music performance self-efficacy (MPSE). MLSE concerns an individual’s belief in their ability to acquire musical skills and knowledge, while MPSE emphasizes their confidence in successfully applying those skills in practice. It is essential to investigate and analyze these two types of music self-efficacy separately.

Many studies suggest that gender may be one of the key factors influencing MSE. Some studies have found that females may exhibit higher levels of self-efficacy during public performances (Zarza-Alzugaray et al., 2020), while others suggest that males may demonstrate stronger music performance self-efficacy (Nielsen, 2004). Overall, the impact of gender on music self-efficacy remains a topic of debate (Clark, 2010; Dempsey and Comeau, 2019; Arbinaga, 2023).

In addition, MSE may vary with academic year, age, and experience. For instance, younger students may exhibit higher self-efficacy due to the relative simplicity of their tasks. However, as learning tasks become more complex, their self-efficacy may decline (Meissner and Timmers, 2020; Bersh, 2021). Conversely, other studies indicate that experienced senior students often display higher self-efficacy when tackling complex tasks compared to their junior counterparts (de Bruin, 2018). For professional musicians, self-efficacy may decrease due to the effect of heightened self-expectations (Spahn et al., 2024).

Furthermore, differences in MSE are observed among learners of different instrument types. For example, students of wind and percussion instruments often report higher self-efficacy in performance preparation compared to string instrument students (Hendricks, 2009).

Overall, the diverse characteristics of MSE highlight the importance of individual differences and underscore the necessity of providing tailored support to meet the varied needs of students.

### 2.2 Music performance anxiety

Among the numerous factors that affect musicians’ performance, music performance anxiety (MPA) has become a central focus for researchers. Due to the demands of their profession, musicians frequently experience the pressure of performing on stage or in front of an audience. MPA is defined as a significant and persistent feeling of anxiety associated with musical performance, often stemming from a combination of biological, psychological vulnerabilities, and situational factors (Osborne and Kenny, 2005; Kenny, 2009). It manifests through emotional, cognitive, physical, and behavioral symptoms, and can arise in a variety of performance contexts, including both solo and ensemble settings.

High levels of MPA are closely associated with reduced concert participation, lower self-efficacy, and poorer academic performance (Barros et al., 2024). MPA not only affects the quality of a musician’s performance but can also have profound implications for their career development (Mazzarolo et al., 2023; Casanova et al., 2025). Many performers report that MPA causes discomfort before or during performances, negatively impacting their stage presence. Research has shown that sustained high levels of MPA may lead musicians to reduce performance opportunities or even consider leaving their musical careers altogether (Spahn et al., 2024).

Research indicates that MPA varies significantly among individuals, affected by factors such as gender (Patston and Osborne, 2016; Hu, 2024), academic year (Hallam et al., 2016; Bersh, 2021), and the distinction between professional and non-professional musicians (Spahn et al., 2024). Gender, as a focal point of research, remains a topic of debate. Some studies have reported that female musicians tend to exhibit higher levels of MPA than males, beginning in late childhood (Osborne and Kenny, 2005; Wehr-Flowers, 2007). However, a study conducted by Paliaukiene et al. (2018) on students at the Lithuanian Academy of Music and Theatre found no significant gender differences in MPA. Similarly, a survey of Chinese music teachers also concluded that gender does not significantly affect MPA (Cui et al., 2024), a finding consistent with Dempsey and Comeau (2019).

In addition to gender, age and performance experience are also considered critical factors affecting MPA (Fernholz et al., 2019). A review of MPA prevalence found that the rates ranged from 16.5 to 60%, with females experiencing higher rate (Fernholz et al., 2019). However, musicians aged 45–50 and above reported lower levels of MPA compared to younger musicians, suggesting that MPA may diminish with age or accumulated performance experience (Zarza-Alzugaray et al., 2017; Fernholz et al., 2019). Similarly, undergraduate music students exhibit higher levels of MPA than professional performers (Papageorgi et al., 2011; Robson and Kenny, 2017). This disparity may stem from professional musicians’ ability to manage MPA more effectively through extensive stage experience, whereas music students, due to their limited experience or lack of coping strategies, are more vulnerable to MPA (Papageorgi et al., 2010; Papageorgi and Welch, 2020; Papageorgi, 2021).

### 2.3 The relationship between music self-efficacy and performance anxiety

The relationship between music self-efficacy (MSE) and music performance anxiety (MPA) has been a key area of research in recent years. Existing studies generally indicate a significant negative correlation between the two. For instance, Hu (2024) used Structural Equation Modeling to demonstrate that higher self-efficacy and motivation are associated with lower levels of MPA. Similarly, studies by Dempsey and Comeau (2019) and Bersh (2021) confirmed this strong negative correlation among adolescents and young musicians. However, most of the literature focuses on the positive role of self-efficacy in alleviating anxiety (Hewitt, 2015), with limited attention to the potential risks associated with high self-efficacy.

If a musician has high self-efficacy, they are likely to set higher personal goals and expectations, which may make them more sensitive to failure. They may perceive even minor errors as unacceptable while striving for higher levels of performance, thereby increasing performance anxiety (Mor et al., 1995; Patston and Osborne, 2016). This suggests that strong self-efficacy may, in some cases, lead to heightened psychological pressure and anxiety, reflecting a positive correlation between self-efficacy and anxiety under specific circumstances.

In the context of mainland China, research has begun to explore the relationship between performance anxiety and self-efficacy among Chinese students and music teachers. Studies by Cui et al. (2024) and Hu (2024) demonstrated a significant negative correlation between self-efficacy and performance anxiety. However, these studies primarily focus on broad groups of music learners and teachers, without delving into differences across specific types of musical instruments. In reality, learners of different instrument types may exhibit varying levels of self-efficacy and performance anxiety.

### 2.4 Research questions

The literature review indicates that students’ gender, academic year, and conservatory level can significantly affect their MLSE, MPSE, and MPA. Gender may affect students’ confidence in both music learning and performance, as well as their performance anxiety. Academic years are associated with experience, expertise, and age, all of which can impact self-efficacy and anxiety, particularly as students’ progress and their expectations become more realistic. Furthermore, conservatory level reflects variations in resources, teaching quality, and performance opportunities, which can also affect students’ self-efficacy and performance.

However, in the previous literature review, it is evident that different scholars have reached inconsistent conclusions regarding the impact of the same factor, with even contradictory findings. These conflicting results may stem from three possible reasons. First, many studies have not focused on specific areas, and different types of music learners may experience varying psychological processes and anxiety depending on the instrument they play. Second, existing studies tend to measure self-efficacy broadly, without differentiating specific contexts. For instance, self-efficacy in music learning focuses on confidence during the learning process, while self-efficacy in music performance is more about coping ability in real performance situations. Mixing these two concepts may lead to inconsistent findings. Finally, differences in the cultural backgrounds of the individuals involved in study may also contribute to the variation in the conclusions. Cultural background may play a key role in moderating psychological experiences and could be a crucial factor affecting research results.

To address the three issues identified, this study focuses on undergraduate violin students in Mainland China (a single instrumental major) to minimize confounding effects from differences across music disciplines, conducting the investigation within a unified cultural context. By distinguishing between two dimensions of self-efficacy—music learning self-efficacy (MLSE) and music performance self-efficacy (MPSE)—we aim to gain a more precise understanding of the relationship between self-efficacy and performance anxiety. The specific research questions (RQs) of this study are as follows:

*RQ1*: What are the effects of gender, academic year, and conservatory level on MLSE, MPSE, and MPA among violin undergraduates in mainland China?

*RQ2*: What is the relationship between MLSE, MPSE, and MPA?

Examining MLSE, MPSE and their connection to MPA can help educators understand students’ emotional states. This insight can inform more targeted teaching and intervention strategies.

## 3 Method

### 3.1 Participants and data collection

The participants are undergraduate violin majors from 11 music conservatories in China, each specializing in music education and training professional musicians. According to China’s university quality evaluation standards, these institutions were categorized into three levels: A-level, B-level, and C-level.^1^ We distributed invitation letters (including consent statements) and online questionnaire links via social messaging apps and email. The invitation included the consent statement: “By clicking the link below, participants indicate their agreement to take part in this study.”

From August to September 2022, the electronic questionnaires were distributed with a snowball sampling method. A total of 345 responses were collected. After excluding 81 responses outside the target population and 10 with completion times under 60 s, 254 valid questionnaires were obtained. The distribution of participants’ gender is almost balanced, with 49.2% male and 50.8% female. In terms of age, 97.6% are between 18 and 25 years old, and 2.4% are under 18. The student distribution by academic year is relatively even, with 20.5% first-year, 25.2% second-year, 31.5% third-year, and 22.8% fourth-year students. The percentages of participants from music conservatory level A, B, and C are 16.9, 43.3, and 39.8%, respectively. Detail demographic information of participants is presented in Table 1.

**Table 1:** Demographic distribution of the participants.

Variables	Category		Frequency	Percentage
Gender	Male		125	49.2%
	Female		129	50.8%
Age	Under 18		6	2.4%
	18–25 years old		248	97.6%
Academic year	Lower-year	First-year	52	20.5%
		Second-year	64	25.2%
	Upper-year	Third-year	80	31.5%
		Fourth-year	58	22.8%
Music conservatory level	Level A		43	16.9%
	Level B		110	43.3%
	Level C		101	39.8%
Total			254	100%

### 3.2 Instruments

For the music self-efficacy scale, this study employed the version developed by Ritchie and Williamon (2010), consisting of two subscales: the music learning self-efficacy subscale (MLSE; 11 items) and the music performance self-efficacy subscale (MPSE; 9 items). Representative items included: “I am confident that I can successfully learn the music for this performance” (MLSE) and “I am confident that I can succeed in the performance” (MPSE). Responses were recorded on a 7-point Likert scale, ranging from 1 (“Strongly Disagree”) to 7 (“Strongly Agree”). The original study reported Cronbach’s *α* coefficients of 0.82 for MLSEs and 0.78 for MPSEs, demonstrating good internal reliability (Ritchie and Williamon, 2010). In the present study, the Cronbach’s alpha coefficients were 0.67 for MLSE and 0.71 for MPSE.

For the music performance anxiety, we used the music performance anxiety inventory for adolescents (MPAI-A), developed by Osborne and Kenny (2005). The MPAI-A, a self-reported measure of music performance anxiety, has been validated for adolescents aged 12–19. It consists of 15 items representing cognitive, physical, and behavioral symptoms of MPA. A representative item includes: “Before I perform, I get butterflies in my stomach.” The scale uses a 7-point Likert scale, where 0 indicates no perceived anxiety symptoms and 6 indicates extreme anxiety (MacAfee and Comeau, 2020). The scale’s Cronbach’s alpha is 0.91 in original study, indicating high internal consistency and reliability (Osborne and Kenny, 2005). In this study, the Cronbach’s alpha coefficient was 0.87.

Since these scales align perfectly with our study’s context, we adopted the original versions directly with permission from the original authors. A master’s graduate in English translation was invited to translate the scales into Chinese, and three experts in violin performance, music education, and educational psychology reviewed the translations. Then five violin students from a music conservatory were invited to read the items to ensure semantic clarity and accurate comprehension. Finally, the translations were back-translated into English by another master’s graduate in English translation, and the back-translated versions were compared with the originals to ensure consistency.

### 3.3 Data analysis

Data analysis in this study was conducted using Python 3.13. Descriptive statistics were first performed on the sample to examine the demographic distribution of participants in terms of gender, age, academic year, and conservatory level. We first conducted a preliminary one-way ANOVA for MLSE, MPSE and MPA. Its results revealed significant differences across academic years (MLSE: *F* (3,250) = 18.34, *p* < 0.001, *η*ₚ^2^ = 0.18; MPSE: *F* (3,250) = 18.66, *p* < 0.001, *η*ₚ^2^ = 0.16; MPA: *F* (3,250) = 8.38, *p* < 0.001, *η*ₚ^2^ = 0.09). Post-hoc tests confirmed similar outcomes between the first and second years, as well as the third and fourth years (see Appendix). As a result, we merged the first with the second year, and the third with the fourth year, reducing the number of groups and increasing the sample size for each group in the subsequent analysis. Based on these results, the first and second years were combined into a “lower-year” group, and the third and fourth years were combined into a “higher-year” group, reducing the academic year levels from four to two.

The frequency distribution across gender, academic year, and conservatory level is presented in Table 2. Since the cross-tabulation indicated that the sample sizes for different gender groups in the upper-year of Level A were too small for a valid three-way ANOVA, gender was analyzed separately as an independent variable to assess its impact on MLSE, MPSE, and MPA. Subsequently, two-way ANOVAs were conducted to examine the effects of conservatory level and academic year on MLSE, MPSE, and MPA.

**Table 2:** Frequency distribution across gender × academic year × conservatory level.

Music conservatory level	Gender	Academic year		Total
		Lower-year	Upper-year	
Level A	Male	18	7	25
	Female	16	2	18
	Total	34	9	43
Level B	Male	14	32	46
	Female	25	39	64
	Total	39	71	110
Level C	Male	22	32	54
	Female	21	26	47
	Total	43	58	101
Total	Male	54	71	125
	Female	62	67	129
	Total	116	138	254

Next, correlation analysis was conducted to explore the relationships between the three variables: MLSE, MPSE, and MPA. Finally, a multiple linear regression was conducted with MPA as the dependent variable and MLSE and MPSE as independent variables to examine their effect on MPA.

## 4 Results

Descriptive statistics were computed for the three main variables with a sample size of 254 and no missing data (Table 3). Analyses of skewness and kurtosis indicated that the distributions of MLSE and MPSE were close to normal, with slight positive skewness. The distribution of MPA exhibited slight negative skewness.

**Table 3:** Descriptive data for MLSE, MPSE and MPA.

Variables	*M*	SD	Range	Skewness	Kurtosis
MLSE	4.37	0.81	2.82–6.36	0.43	−0.70
MPSE	4.31	0.75	2.44–7.00	0.53	0.24
MPA	5.03	0.86	2.27–6.80	−0.24	−0.78

### 4.1 Differences in MLSE

An independent samples t-test revealed no significant gender difference in MLSE, *t* (251) = −1.781, *p* = 0.076, *d* = −0.223, 95% CI [−0.382, 0.020]. Female students (*M* = 4.46, SD = 0.81) showed marginally higher scores than male students (*M* = 4.28, SD = 0.79), but this difference did not reach statistical significance.

A two-way ANOVA was employed to examine the main effects and interactions of academic year (lower-year, upper-year), and conservatory level (A, B, C) on MLSE. Levene’s test indicated homogeneity of variances (*W* = 2.03, *p* = 0.075). Although the variable exhibited slight skewness, ANOVA is robust to violations of the normality assumption (Brown and Forsythe, 1974; Fidell et al., 2013; Knief and Forstmeier, 2021), and therefore, it remains appropriate for this analysis.

The two-way ANOVA revealed a significant main effect of conservatory level, *F* (2, 248) = 13.65, *p* < 0.001, *η*_p_^2^ = 0.077, 95% CI [0.032, 0.147]. Games-Howell post-hoc tests showed significant differences between Level A (*M* = 4.93, SD = 0.86) and Level B (*M* = 4.27, SD = 0.87, *p* < 0.001, Hedges’ *g* = 0.76), as well as between Level A and Level C (*M* = 4.24, SD = 0.59, *p* < 0.001, Hedges’ *g* = 1.02), but not between Levels B and C (*p* = 0.933). The results indicate that students’ MLSE in Level A conservatories was significantly higher than in both Level B and Level C conservatories, with no significant difference between the latter two.

**Figure 1:**
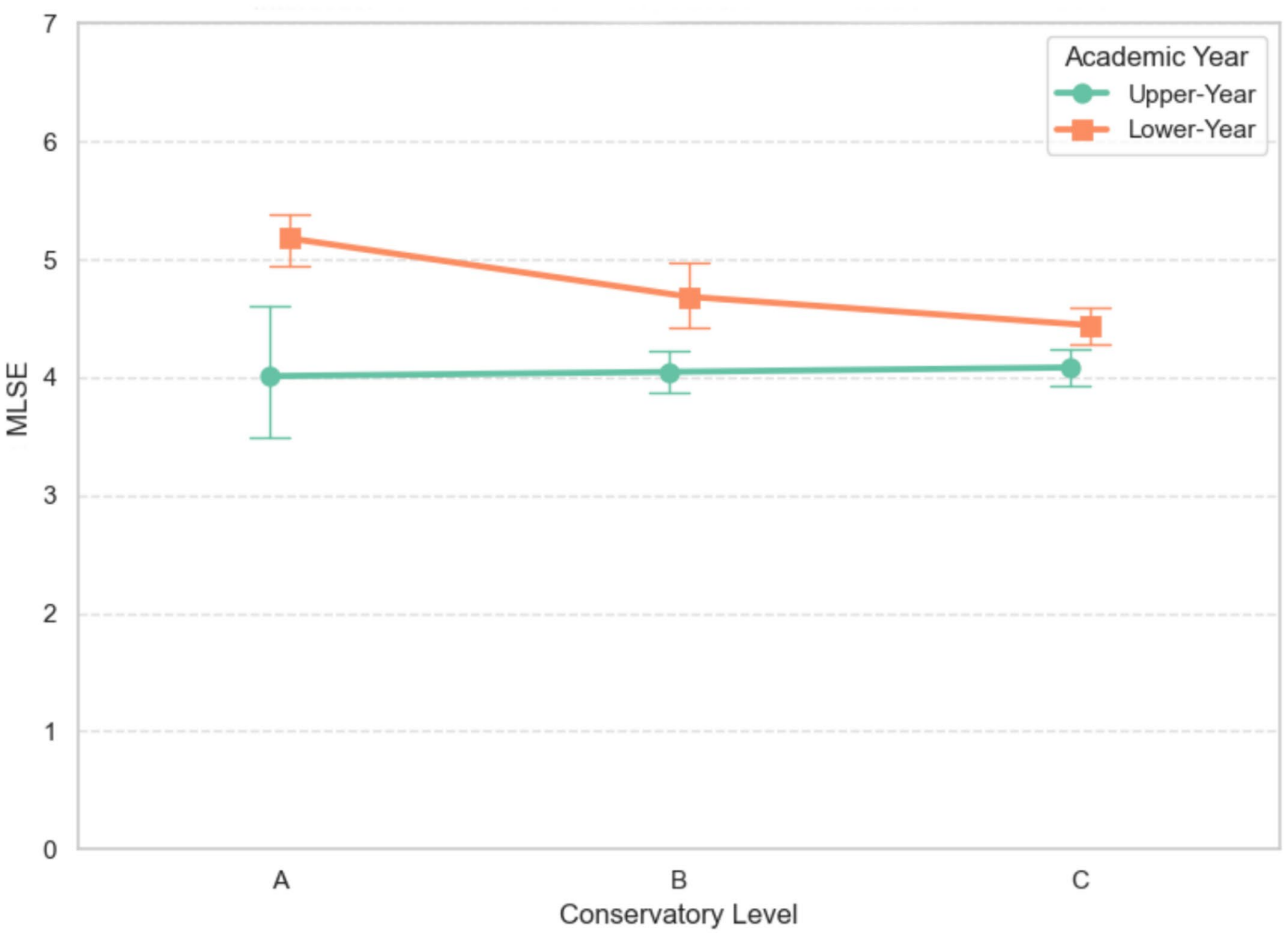
Two-way interaction plot of academic year × conservatory level on MLSE.

There was also a significant main effect of academic year (*F* (1, 248) = 11.32, *p* = 0.001, *η*_p_^2^ = 0.072, 95% CI [0.010, 0.170]), with lower-year students (*M* = 4.74, SD = 0.76) showing higher MLSE than upper-year students (*M* = 4.06, SD = 0.71, *p* < 0.001, Hedges’ *g* = 0.92). The interaction between conservatory level and academic year was not statistically significant (*F* (2, 248) = 2.96, *p* = 0.054, *η*_p_^2^ = 0.029, 95% CI [0.004, 0.098]). The two-way interaction plot on MLSE is presented in Figure 1.

### 4.2 Differences in MPSE

No significant gender difference was found in MPSE, *t* (251) = −0.825, *p* = 0.410, *d* = −0.104, 95% CI [−0.270, 0.105]. Female students (*M* = 4.35, SD = 0.77) and male students (*M* = 4.27, SD = 0.74) showed similar levels of music performance self-efficacy.

To ensure the validity of subsequent ANOVA, the homogeneity of variance assumption was examined first. Levene’s test indicated heterogeneity of variances (*W* = 3.13, *p* = 0.009). A robust two-way ANOVA with HC3 standard errors was used to address violation of homogeneity assumptions. The result showed a significant main effect of conservatory level, *F* (2, 248) = 15.28, *p* < 0.001, *η*_p_^2^ = 0.083, 95% CI [0.036, 0.160]. Games-Howell post-hoc tests revealed significant differences between Level A (*M* = 4.93, SD = 0.68) and Level B (*M* = 4.25, SD = 0.80, *p* < 0.001, Hedges’ *g* = 0.89), between Level A and Level C (*M* = 4.10, SD = 0.57, *p* < 0.001, Hedges’ *g* = 1.36), but not between Levels B and C (*p* = 0.276). The results indicate that students’ MPSE in Level A conservatories was significantly higher than in both Level B and Level C conservatories, with no significant difference between the latter two.

**Figure 2:**
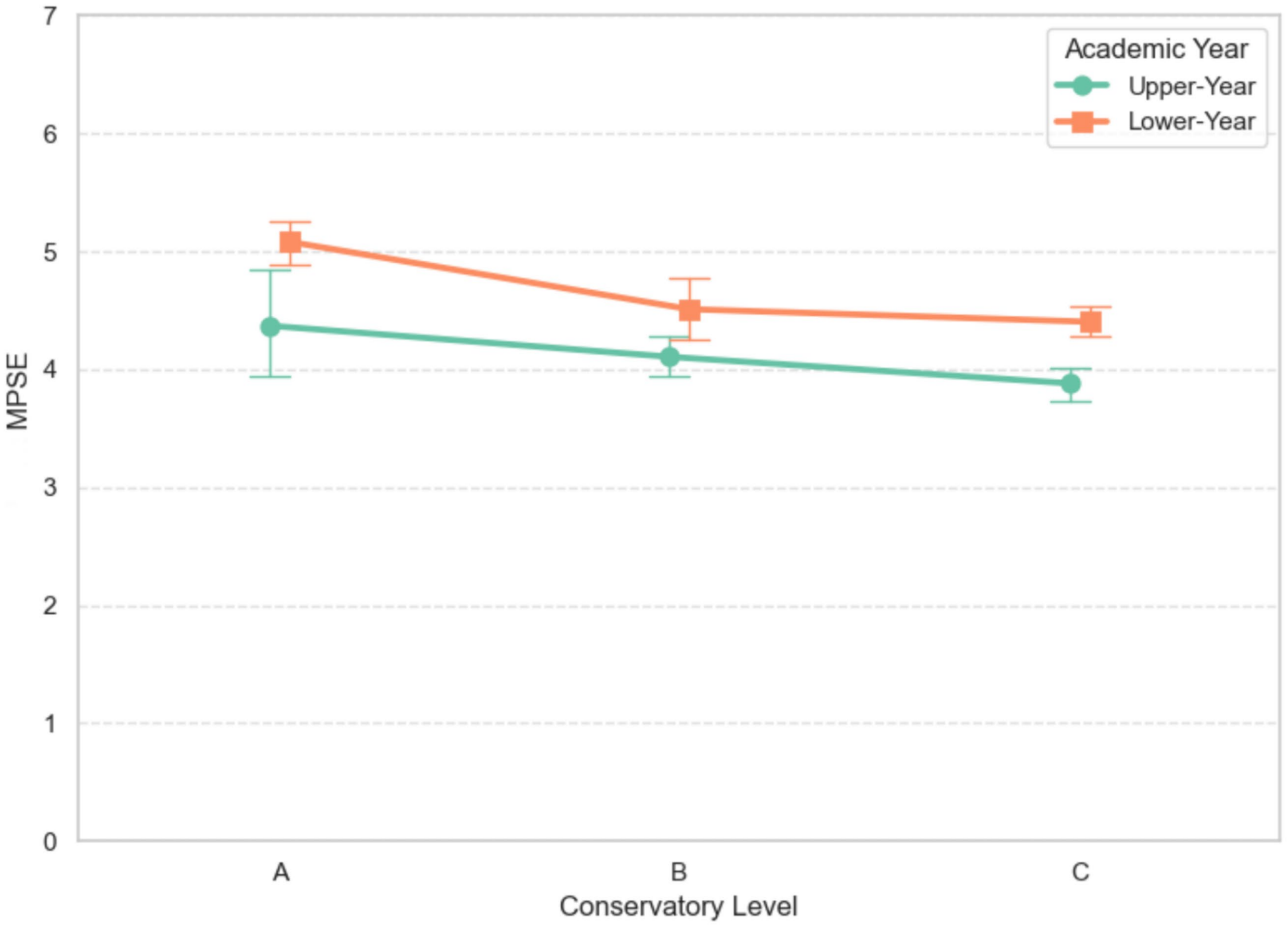
Two-way interaction plot of academic year × conservatory level on MPSE.

A significant main effect of academic year was also found, *F* (1, 248) = 6.24, *p* = 0.013, *η*_p_^2^ = 0.033, 95% CI [0.003, 0.097], with lower-year students (*M* = 4.64, SD = 0.71) reporting higher MPSE than upper-year students (*M* = 4.03, SD = 0.67, *p* < 0.001, Hedges’ *g* = 0.88). The interaction effect was not significant, *F* (2, 248) = 0.46, *p* = 0.629, *η*_p_^2^ = 0.005, 95% CI [0.000, 0.052]. The two-way interaction plot on MPSE is presented in Figure 2.

### 4.3 Differences in MPA

The gender difference in music performance anxiety (MPA) was not statistically significant, *t* (251) = −1.560, *p* = 0.120, with a small effect size (*d* = −0.196, 95% CI [−0.371, 0.044]). Although female students reported marginally higher anxiety levels (*M* = 5.12, SD = 0.89) than their male counterparts (*M* = 4.95, SD = 0.83), this difference did not reach statistical significance.

The assumption of homogeneity of variance was first tested. Levene’s test indicated heterogeneity of variances (*W* = 4.73, *p* < 0.001). A robust two-way ANOVA revealed a significant main effect of conservatory level (*F* (2, 248) = 9.30, *p* < 0.001, *η*_p_^2^ = 0.086, 95% CI [0.027, 0.169]). Games-Howell post-hoc tests showed significant differences between Level A (*M* = 5.29, SD = 0.89) and Level B (*M* = 4.72, SD = 0.83, *p* = 0.002, Hedges’ *g* = 0.67), as well as between Level B and Level C (*M* = 5.27, SD = 0.78, *p* < 0.001, Hedges’ *g* = −0.67), but not between Levels A and C (*p* = 0.988).

The main effect of academic year approached but did not reach significance^2^ (*F* (1, 248) = 3.57, *p* = 0.060, *η*_p_^2^ = 0.022, 95% CI [0.000, 0.083]). Although non-significant, the effect size suggests 2.2% of MPA variance was associated with academic year. Descriptive statistics showed lower-year students (*M* = 5.31, SD = 0.89) reported higher levels than upper-year students (*M* = 4.80, SD = 0.76), with a mean difference of 0.52 points.

**Figure 3:**
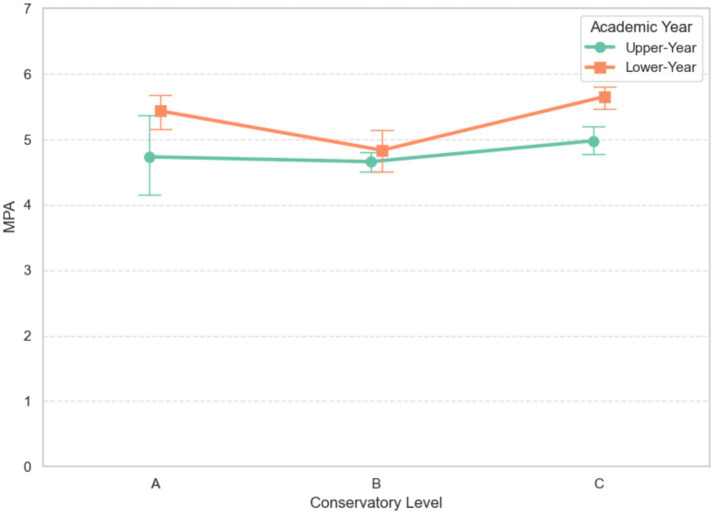
Two-way interaction plot of academic year × conservatory level on MPA.

The interaction effect was also not significant (*F* (2, 248) = 2.44, *p* = 0.089, *η*_p_^2^ = 0.023, 95% CI [0.002, 0.086]). The two-way interaction plot on MPA is presented in Figure 3.

### 4.4 Correlation analysis of MLSE, MPSE and MPA

The Pearson correlation analysis revealed significant relationships among MLSE, MPSE and MPA. A statically significant correlation was found between MLSE and MPSE (*r* = 0.73, *p* < 0.01). Additionally, MLSE and MPA exhibited a moderate statistically significant correlation (*r* = 0.40, *p* < 0.01), while the correlation between MPSE and MPA was weaker, though still significant (*r* = 0.18, *p* < 0 0.01). This finding aligns with the analyses presented in Sections 4.1 and 4.2.

### 4.5 Multiple regression analysis

Focusing on MPA as the dependent variable, a linear regression analysis was conducted to explore its relationship with the mean scores of MLSE and MPSE as independent variables. To improve the accuracy of the regression model, demographic variables (gender, academic year and conservatory level) were included as control variables. The first level of each control variable is used as the reference level.

The regression model demonstrated a good overall fit, with an R-squared of 0.283 and an adjusted R-squared of 0.269. The F-statistic was 19.59 (df = 5, 248), *p* < 0.001, suggesting that the model is statistically significant. The Variance Inflation Factors (VIFs) for all variables were below 3 (Table 4), indicating that multicollinearity did not pose a significant issue.

**Table 4:** Multiple linear regression results for MPA.

Variables	Model	Non-standardized coefficients		Standard coefficients	*t*	sig	VIF
		*B*	SE (*B*)	*β*			
Independent variable	Constant	4.03	0.40				
	MLSE	0.53	0.09	0.50	6.20	<0.001	2.24
	MPSE	−0.27	0.09	−0.23	−2.83	0.005	2.33
Controlled variables	Male (reference category)						
	Female	0.14	0.09	0.08	1.44	0.15	1.04
	Lower-year (reference category)						
	Upper-year	−0.28	0.11	−0.16	−2.63	0.009	1.29
	Conservatory Level A (reference category)						
	Conservatory Level B	−0.27	0.14	−0.16	−1.92	0.056	2.36
	Conservatory Level C	0.23	0.15	0.13	1.57	0.117	2.40

The regression analysis revealed that, after controlling for demographic factors, MLSE had a significant positive effect on MPA (*B* = 0.53, *p* < 0.01), suggesting that higher MLSE might be associated with greater MPA, potentially due to higher personal expectations and self-imposed pressure. In contrast, MPSE exhibited a significant negative effect on MPA (*B* = −0.27, *p* < 0.01), indicating that higher MPSE effectively reduces performance anxiety, reflecting greater confidence and emotional stability during performances.

## 5 Discussion

This study aims to examine the music learning self-efficacy (MLSE), music performance self-efficacy (MPSE), and music performance anxiety (MPA) of undergraduate violin students in mainland China, with a focus on gender, academic year, and conservatory level. Furthermore, the study investigates the relationships between MLSE, MPSE, and MPA.

### 5.1 Differences in MSE

This study found no significant gender differences in MLSE or MPSE among undergraduate violin majors in mainland China. Previous research has shown that gender can play a significant role in self-efficacy in different academic or professional contexts (Huang, 2012; Mayra, 2022). Other studies have reported gender differences in areas such as music preferences, instrument selection, and cultural norms (Colley et al., 1994; O’Neill and Boultona, 1996; Ho, 2006). However, these gender differences were not observed in the self-efficacy levels of violin majors in mainland China.

Compared to previous studies, this research specifically focused on undergraduate students within the same major (violin), effectively controlling for gender-related biases caused by differences in academic or program contexts. This not only enhances the reliability of the findings but also further confirms that gender is not a determining factor in MLSE or MPSE.

The main effect of academic year on MLSE was significant, with lower-year students showing notably higher MLSE compared to their upper-year counterparts. This result may reflect the psychological changes that students experience during their undergraduate years. Lower-year students often display heightened self-efficacy due to their enthusiasm and the novelty of studying at a conservatory. Additionally, lower-year students benefit from teaching approaches focused on skill development and experience-building, such as one-on-one lessons and group practice (Bandura, 1982), which play a vital role in fostering and enhancing self-efficacy. However, as students’ progress in their studies, upper-year students tend to face increased academic demands and technical challenges, which may lead to a decline in self-efficacy. Moreover, upper-year students are often burdened with tasks like career planning, internships, and graduation requirements, which may reduce their focus on music study and, in turn, lower their confidence in learning.

The study also found that conservatory level had a significant main effect on both MLSE and MPSE. Students from A-level conservatories demonstrated significantly higher levels of MLSE and MPSE compared to students from B- and C-level conservatories. This disparity may be attributed to differences in admissions standards and talent development models among these conservatories.

A-level conservatories have higher admission requirements for professional skills and artistic potential. Students admitted to these conservatories typically receive systematic instrumental training and music theory education from a young age, entering college with solid foundational skills and advanced musical literacy (Pajares, 1996). This strong background enables them to master complex musical techniques and theoretical knowledge, resulting in significantly higher self-efficacy in music learning and performance compared to peers from B- and C-level conservatories.

Regarding talent development models, A-level conservatories focus on training performance-oriented musicians for professional artistic ensembles, offering a curriculum centered on high-level musical performance, practical opportunities, and intensive professional training (Tang, 2010). In contrast, B- and C-level conservatories emphasize producing music educators, with curricula prioritizing pedagogy and basic music theory, often with less focus on stage performance (Zhu, 2013; Wu, 2020). These differences in educational objectives may affect students’ MLSE and MPSE.

### 5.2 Differences in MPA

This study shows that the MPA of undergraduate violin students in mainland China is 5.03, indicating a relatively high level on a 0–6 scale, with no significant gender differences observed. Some studies have reported that female musicians, particularly students and professional performers, tend to report higher levels of MPA (Kenny, 2011; Dobos et al., 2018; Barros et al., 2024). Contrary to these findings, our study reveals that the MPA of undergraduate violin students do not exhibit gender differences. This aligns with a study conducted at the Lithuanian Academy of Music and Theatre, where Paliaukiene et al. (2018) found no significant relationship between gender and MPA. Similarly, a study on MPA among Chinese music teachers reported comparable results (Cui et al., 2024). It suggested that the lack of gender differences might be due to a severe gender imbalance in the sample. In contrast, our study’s nearly balance gender ratio eliminates this confounding factor, providing stronger evidence that gender does not significantly impact MPA levels for undergraduate violin students in mainland China.

Our analysis revealed no statistically significant difference in MPA between lower-year and upper-year violin students in Chinese conservatories. While some previous research has suggested that senior students experience higher MPA due to professional pressures (Studer et al., 2011; Kenny et al., 2012), our findings showed a different tendency. This non-significant trend aligns better with studies highlighting the transitional challenges faced by early-year students (Papageorgi et al., 2007; Zarza-Alzugaray et al., 2017), who must adapt to new academic environments while developing performance skills.

An intriguing finding in our study is that MPA does not follow a linear relationship with conservatory level but instead exhibits a U-shaped pattern. Students from A- and C-level conservatories reported higher MPA compared to those from B-level conservatories. This could be due to A-level conservatory students facing intense competition and high self-expectations, which may lead to anxiety driven by an excessive focus on outcomes. On the other hand, students from C-level conservatories might experience increased anxiety due to limited resources, fewer opportunities, and uncertainty about their future. In contrast, B-level conservatories seem to offer a more balanced mix of resources and competition, contributing to a more balanced mindset and lower anxiety levels among students.

Our study extends the research of Papageorgi et al. (2007) and Zarza-Alzugaray et al. (2017). Papageorgi’s study compared the MPA levels between classical musicians and jazz musicians, while Zarza-Alzugaray’s research mainly focused on comparing the MPA levels between music schools with a predominance of amateur students and career-oriented higher music institutions. In contrast, our study compares the MPA levels of students from three different conservatory levels within the same instrument type. Through this comparison, our research provides a more detailed perspective, revealing the potential impact of conservatory level on students’ MPA.

### 5.3 Relationship between MPA and MLSE, MPSE

After controlling for the variables of gender, academic year and conservatory level, our study found that MLSE and MPSE have distinct effects on MPA among undergraduate violin students in China. High MLSE is associated with higher levels of MPA, whereas high MPSE effectively reduces MPA.

MLSE reflects a performer’s belief in their ability to acquire the skills necessary to master a musical piece (Ritchie and Williamon, 2011). While high MLSE is associated with positive learning motivation and better performance outcomes (Wang, 2025), it can also lead to heightened self-expectations and stress. Violin students with high MLSE may set high performance standards, striving for both technical accuracy and artistic expression (Yang, 2023; Di Stefano et al., 2024). This can result in anxiety.

In contrast, MPSE—defined as the performer’s belief in their ability to deliver a complete and successful musical performance (Ritchie and Williamon, 2010)—is effective in reducing MPA. In formal performance settings, violinists often face the pressure of audience scrutiny. Those with high MPSE are better able to manage their emotions, stay composed under pressure, and quickly recover from mistakes without letting disruptions affect the rest of their performance. This emotional regulation capability mitigates anxiety (MacAfee and Comeau, 2020; Zelenak, 2024). Additionally, MPSE is also derived from prior performance experiences (Kleppang et al., 2023). Each successful performance reinforces self-efficacy, making performers more confident when facing future performances. This creates a positive feedback loop that effectively reduces performance anxiety.

The relationship between MPSE and MPA reveals a suppression effect. Although the simple correlation between MPSE and MPA is positive and significant, the strength of this relationship is relatively mild. After controlling for MLSE and other demographic variables, regression analysis shows a negative independent effect of MPSE on MPA. This shift in direction suggests that the true relationship between MPSE and MPA is masked by the influence of MLSE. The strong correlation between MLSE and MPSE likely contributes to this effect, as MPSE shares a significant amount of variance with MLSE.

## 6 Implication

The research findings further suggest that MLSE and MPSE of violin students are distinct from each other, although there is a moderate to strong statistically significant correlation between the two. MLSE emphasizes confidence in mastering musical knowledge and skills, focusing on the perceived ability during the learning process (Orejudo et al., 2021; Casanova et al., 2022; Chen, 2024; Su et al., 2024). It reflects students’ confidence in tackling learning tasks and overcoming challenges (Ritchie and Williamon, 2011). In contrast, MPSE refers to confidence in one’s performance abilities during actual playing, encompassing technical skills, expressive capabilities, and the ability to handle performance-related pressure and situational factors (e.g., audience reactions, stage environment) (Ritchie and Williamon, 2010; Zelenak, 2020; Zarza-Alzugaray et al., 2020). Therefore, while there is a certain correlation between MLSE and MPSE, they differ significantly in their underlying meanings and functions.

This finding has important implications for music educators. When educators aim to reduce performance anxiety in violin students, merely enhancing students’ MLSE may not necessarily alleviate their performance anxiety, as the sources of anxiety in these two areas are different. MLSE focuses more on self-perception during the learning process, while MPA often arises in actual performance situations (Zarza-Alzugaray et al., 2020; Tahirbegi, 2021). Therefore, mitigating MPA may require more direct efforts to enhance students’ confidence and sense of competence during performances. In contrast, improving students’ MPSE, especially their ability to cope with high-pressure situations, technical proficiency, and emotional expression during performances, may be more effective in helping students reduce MPA and improve their performance outcomes.

Therefore, music educators should focus on developing students’ MPSE during the learning process. Techniques such as scenario simulations, stress management training, and performance practice can help students build confidence in real performance settings, enabling them to better cope with performance anxiety and enhance the quality of their performances. Additionally, understanding the differences between MLSE and MPSE will help educators create more personalized and targeted teaching strategies, thereby more effectively assisting students in overcoming psychological barriers and achieving artistic growth.

## 7 Limitations and future research

This study has the following three limitations. First, while we aim to provide useful references for music students influenced by Chinese culture and support relevant theories that represent a broader population, the sample in our study primarily focuses on undergraduate violin students. The data was collected from 11 music conservatories in China, but it does not cover all 14 music conservatories in mainland China, nor does it include violin students from comprehensive universities with music departments. This limitation can be seen as a constraint in sampling and data collection frequency, which may affect the generalizability of the research conclusions.

Second, we employed a cross-sectional survey design, capturing data at a single time point. However, self-efficacy and performance anxiety may fluctuate over time. For instance, anxiety levels tend to increase before exams or performances, which might introduce bias into our conclusions.

Third, our study relies on students’ self-reports, which may lead to response bias. Participants might overestimate or underestimate their self-efficacy or performance anxiety, potentially affecting the accuracy of the research findings.

Future research would benefit from extending the current study in several directions. First, expanding the participants to include a wider range of music institutions and cultural backgrounds would enhance the generalizability of the findings. Second, implementing longitudinal designs could provide valuable insights into the developmental patterns of MSE and MPA. Third, incorporating mixed methods approaches such as qualitative interviews and behavioral observations would help address potential limitations of self-report data. Furthermore, future studies would explore the mechanisms behind the suppression effect between MPSE and MPA, focusing on the moderating role of MLSE.

## 8 Conclusion

Musical self-efficacy and performance anxiety are critical topics for music educators. This study revealed that both MLSE and MPSE were at medium levels, with no significant gender differences. However, academic year differences were observed in both MLSE and MPSE, with lower-year students reporting significantly higher MLSE and MPSE than upper-year students. Additionally, differences were found based on conservatory level, with students from top-tier (A-level) conservatories reporting higher MLSE and MPSE than their peers from B- and C-level conservatories.

Chinese undergraduate violin students exhibited relatively high MPA without gender differences. No significant difference in MPA was found between lower-year and upper-year students. A U-shaped distribution in MPA was observed across conservatory rankings, with A- and C-level conservatory students showing significantly higher MPA than those from B-level conservatories. MLSE positively predicted MPA, while MPSE showed a negative relationship with MPA.

These findings suggest that MLSE and MPSE are distinct constructs for violin students, despite their moderate-to-high correlation. This suggests that solely enhancing MLSE may not effectively reduce MPA in violin students, whereas improving MPSE is likely to be more beneficial in alleviating performance anxiety.

## Data Availability

The data that support the findings of this study are available from the corresponding author upon reasonable request.

## Footnotes

^1^The music conservatory rankings here are based on the 2022 China Music University Ranking by the Alumni Association, which uses a comprehensive evaluation system covering teaching quality, distinguished alumni, research achievements, high-level talents, and other core indicators. The rankings also consider academic disciplines, social reputation, and international influence, with scores calculated through a weighted system. The rankings can be accessed at: http://www.cuaa.net/.

^2^The preliminary one-way ANOVA demonstrated significant differences in MPA between year groups (see Appendix), with post-hoc analyses confirming higher MPA levels in lower-year students compared to upper-year students. However, this effect was no longer significant in the two-way ANOVA, likely due to variance redistribution when including the second independent variable, which accounted for portions of variance originally associated with the year-group distinction.

## Appendix: One-way ANOVA for MLSE, MPSE, MPA by academic year

**Table:**

Dimension	Academic year	*n*	Mean	SD	Sig				Result
					1	2	3	4	
MLSE	1	52	4.82	0.86		0.906	<0.001	<0.001	1, 2 > 3, 4
	2	64	4.68	0.67			<0.001	<0.001	
	3	80	4.06	0.66				1.000	
	4	58	4.05	0.78					
MPSE	1	52	4.70	0.80		1.000	<0.001	0.001	1, 2 > 3, 4
	2	64	4.59	0.63			<0.001	0.008	
	3	80	3.92	0.63				0.145	
	4	58	4.18	0.69					
MPA	1	52	5.28	0.87		1.000	0.017	0.004	1, 2 > 3, 4
	2	64	5.34	0.92			0.002	0.001	
	3	80	4.84	0.75				1.000	
	4	58	4.74	0.78					
